# Tecovirimat use among patients with monkeypox (mpox) in Alameda County, California, June–October 2022

**DOI:** 10.1017/ash.2023.379

**Published:** 2023-09-29

**Authors:** Megan Ouyang, Munira Shemsu, Rachel Marusinec, April Pena, Kavita Trivedi, Eileen Dunne, Emily Yette, Nicholas Moss, Paul Bayard, Magdalen Edmunds, Sunny Lai, Mychi Nguyen, Sumanth Rajagopal, Sally Slome, Michele Tang, George Ayala, Amit Chitnis

## Abstract

**Background:** Tecovirimat (TPOXX) is an antiviral drug only available via an Expanded Access Program (EAP) investigational new drug protocol and is recommended for treatment of select patients with monkeypox (mpox) infection. Alameda County Public Health Department prioritizes health equity but does not have a dedicated public health clinic. Therefore, we partnered closely with local healthcare providers that serve communities disproportionally impacted by mpox to ensure there was access to TPOXX. Using data collected during the outbreak we assessed whether populations in Alameda County most affected by mpox received treatment. **Methods:** We describe Alameda County patients with confirmed or probable mpox who received TPOXX during June–October 2022. Data were collected from case investigation interviews with patients and state-wide reportable disease database(s), which included demographic, clinical, and behavioral information. Confidence intervals (CIs) were calculated using the exact method for Poisson counts. We compared characteristics of mpox patients who received and did not receive TPOXX using the Pearson χ^2^ or Fisher exact test. *P* < .05 was considered significant. **Results:** Mpox case rates in Alameda County were highest among Black or African-American residents (35.6 per 100,000, 95% CI, 26.7–46.4) and Hispanic or Latinx residents (25.2, 95% CI, 20.2–31.0) compared to Asian residents (3.9, 95% CI, 2.3–6.1) and white residents (10.4, 95% CI, 7.7–13.9) residents. Among 242 mpox patients, 69 patients (28.5%) received TPOXX. The distribution of demographic and clinical characteristics among patients who received TPOXX was not significantly different than among those who did not, including residents aged 31–40 years (36.2% vs 34.7%), Black or African-American residents (20% vs 26.3%), Hispanic or Latinx residents (38.5% vs 41%), male residents (89.9% vs 95.3%), gay, lesbian, or same-gender loving residents (67.2% vs 67.4%) in the city of Oakland (63.2% vs 61.5%), or residents with human immunodeficiency virus infection (43.5% vs 36.6%). **Conclusions:** During the Alameda County mpox outbreak, nearly one-third of patients received TPOXX. Demographic and clinical characteristics were similar among TPOXX recipients and nonrecipients. A proactive approach to obtaining TPOXX in Alameda County and strong relationships with local providers may have allowed for treatment to be accessible to mpox patients. Regular review of outbreak data can inform public health activities, ensure health equity, and help refine local response efforts.

**Disclosures:** None

